# Matrix metalloproteinases and tissue inhibitors in multiple myeloma: promote or inhibit?

**DOI:** 10.3389/fonc.2023.1127407

**Published:** 2023-09-26

**Authors:** Yan-Ying Li, Liu-Yun Zhang, Yun-Hui Xiang, Dan Li, Juan Zhang

**Affiliations:** ^1^ School of Medicine, University of Electronic Science and Technology of China, Chengdu, China; ^2^ Department of Laboratory Medicine, Sichuan Provincial Key Laboratory for Human Disease Gene Study, Sichuan Provincial People's Hospital, School of Medicine, University of Electronic Science and Technology of China, Chengdu, China; ^3^ School of Laboratory Medicine, Chengdu Medical College, Chengdu, Sichuan, China

**Keywords:** matrix metalloproteinase, tissue inhibitor of metalloproteinases, multiple myeloma, osteolytic bone disease, neoplasm metastasis

## Abstract

Matrix metalloproteinases (MMPs) and tissue inhibitor of metalloproteinases (TIMPs) play a vital role in the pathogenesis of multiple myeloma (MM), especially for tumor invasion and osteolytic osteopathy. By breaking down extracellular matrix (ECM) components and releasing the proteins composing the ECM and growth factors, as well as their receptors, MMPs affect tissue integrity and promote cancer cell invasion and metastasis. A vital pathophysiological characteristic of MM is the progress of osteolytic lesions, which are brought on by interactions between myeloma cells and the bone marrow microenvironment. MMPs, certainly, are one of the fundamental causes of myeloma bone disease due to their ability to degrade various types of collagens. TIMPs, as important regulators of MMP hydrolysis or activation, also participate in the occurrence and evolution of MM and the formation of bone disease. This review focuses on the role of MMP-1, MMP-2, MMP-7, MMP-9, MMP-13, MMP-14, and MMP-15 and the four types of TIMPs in the invasion of myeloma cells, angiogenesis, osteolytic osteopathy, to offer some novel perspectives on the clinical diagnostics and therapeutics of MM.

## 1 Introduction

Abnormal plasma cell clones distinguish MM, the second most common hematologic malignancy after non-Hodgkin’s lymphoma. The pre-disease stage is often asymptomatic but exhibits genetic abnormalities, including hyperdiploidy and translocations involving immunoglobulin heavy chains. This manifests as either monoclonal gammopathy of undetermined significance (MGUS) or smoldering multiple myeloma (SMM) (1). As the disease progresses, clonal proliferation of malignant plasma cells (PCs) in the bone marrow (BM) leads to anemia, myelosuppression, and bone destruction, as well as harm to the kidneys and other organs due to hyperglobulinemia (2). The initiation of extramedullary disease and bone destruction is based on that MM cells stop responding to the BM’s growth and survival signals and migrate into the peripheral blood (3). Malignant cell migration and bone degeneration are both critically dependent on MMPs. In 1962, researchers discovered the first MMP from the tail of a tadpole, which can degrade collagen. In addition to the ability of MMPs to remodel the ECM, MMPs have been shown in recent years to play biological roles in cell signaling, immunological function, and transcriptional regulation (4). They function as regulators of tumor and host interactions because they can change the activity of several cytokines and growth factors (5).

In recent decades, there have been a considerable number of studies on the role of MMPs in malignant tumors, but the majority of previous research mainly focused on the association of MMPs and solid tumor invasion, metastasis and angiogenesis, inflammatory response, and so on, whereas studies on the link between hematological malignancies and MMPs only account for a small part. Here, we mainly review the structures and functions of MMPs and TIMPs, what is more important, the role of MMPs and TIMPs in the progression of MM and the latest advances will be emphasized.

## 2 The structure and function of MMPs and TIMPs

The human MMP family consists of 23 members, each of which exhibits a variety of domains and architectures (**Supplementary Figure 1**). According to its structure, they are classified as archetypal MMPs, gelatinases, matrilysins, convertase-activable MMPs, membrane-type MMPs (MT-MMPs), and MMP-23B (4, 6). MMPs are generally classified into collagenases, gelatinases, stromelysin, matrilysin, membrane-type MMPs, and other MMPs based on their substrates and functions (7–9). Collagenases (MMP-1, MMP-8, MMP-13, and MMP-18) cleave interstitial collagens (types I, II, and III), as well as type IV collagen and type XI collagen (10–13). Additionally, they can degrade various other extracellular matrix (ECM) molecules and non-ECM molecules (12). Gelatinases (MMP-2 and MMP-9) cleave gelatin because of their specific three fibronectin repeats and also degrade ECM (6). Matrilysins (MMP-7 and MMP-26) are unique as they lack a hemopexin domain common to other MMPs. They can cleave both collagen and gelatin *in vitro (* 4). Stromelysins (MMP-3, MMP-10, MMP-11) and collagenases have similar molecular structural domains, but stromelysins can’t cleave interstitial collagen (4). MT-MMPs comprise a transmembrane domain or glycolphosphatidyl inositol (GPI) anchored proteins (6). The pro-domain of MT-MMPs is processed to activate the enzyme intracellularly. They are further directed to the cell surface by virtue of its membrane anchoring domain (14, 15). The six subtypes can be divided into two groups according to their structures: MMP-14, MMP-15, MMP-16, and MMP-24contain type I transmembrane proteins. On the other hand, MMP-17 and MMP-25 have GPI-anchored proteins (**Supplementary Figure 1**). There are seven MMPs (MMP-12, -19, -20, -21,-23 -27, -28) that are not classified in the above categories because of their divergence in sequence and substrate specificity (11).

The four TIMPs show differences in their glycosylation degree, however, mammalian TIMPs show fundamental structural similarities, which fold to constitute a wedge-shaped appearance made up of two major domains (15, 16). Each domain mediates a separate function, with the first having an N-terminal domain of around 125 amino acids and the second having a C-terminal domain of about 65 amino acids (17, 18). The N-terminal domain can form a structure that interacts with the catalytic site of the metalloproteinase, which is essential to inhibit the activity of the metalloproteinase (19). Instead of inhibiting metalloproteinases, the TIMPs’ C-terminal domain primarily mediates their interactions with other proteins (16). The fundamental structure of TIMPs determines their classical function, which is to inhibit MMPs and other related enzymes, such as the disintegrin and metalloproteinases (ADAMs) and ADAMs with thrombospondin motifs (ADAMTSs) (20). Some evidence has shown that ADAM-9 and ADAMTS-9 are aberrantly expressed in malignant plasma cells. The expression of ADAM23 is associated with poor prognosis. Moreover, the cleavage products of ADAM10 and ADAM17, the C-X3-C motif chemokine ligand 1 (CX3CL1)/fractalkine, play a role in inflammation and angiogenesis within the tumor microenvironment. CX3CL1 exhibits elevated levels in the bone marrow of MM patients and represents a novel participant in the MM microenvironment involved in MM-induced angiogenesis (21–23).

## 3 The role of MMPs in MM

It is well known that ECM is a critical component for maintaining tissue and organ structure and function, playing an essential role in the survival of organisms. Meanwhile, changes in ECM composition and structure can lead to the development of many diseases, especially in the context of tumor initiation and progression, where ECM alterations are involved in many critical steps of tumor metastasis (24). MMPs are expressed in a variety of tissues in the normal human body and are widely recognized for their proteolytic function, but subsequently, other functions of MMPs have gradually been discovered and their role in MM has also been studied. In addition to their expression in normal tissues, MMPs are pathologically expressed in the MM BM microenvironment and are involved in the initiation and progression of MM through multiple pathways; these MMPs include MMP-1, MMP-2, MMP-7, MMP-9, MMP-13, MMP-14 and MMP-15 (**Supplementary Figure 2**).

### 3.1 MMP-1 in MM

MMP-1 is one of the prototypical MMPs and is expressed in a wide variety of tissues in the human body, including the liver, kidney, intestine, stomach, placenta, bladder, and pancreas, in pathological conditions, it increases the bioavailability of insulin-like growth factor-1 (IGF-1), promotes cell proliferation and migration, epithelial regeneration, and inflammation, and has a proteolytic activity that degrades physical barriers and promotes cancer progression (25). Previous research suggested that MMP-1 stimulates protease-activated receptor (PAR) 1 by cleaving the same Arg-Ser link that thrombins cleave, which encourages breast cancer cell proliferation and invasion (26).

The primary structural protein of bone is collagen I, and the onset of bone resorption depends on the degradation of this protein. Type I collagen is known to be degraded by MMP-1 at neutral pH levels. Results from an earlier study showed that MM patients have intrinsically high levels of MMP1, and co-culturing them with RPMI8226 cells makes this phenomenon more obvious (27). However, the exact mechanism by which MMP-1 contributes to bone deterioration is still unknown. Another study (28) established that osteoblasts induced myeloma cells to release MMP-1, urokinase plasminogen activator (uPA), and hepatocyte growth factor (HGF); conversely, contact with myeloma cells caused osteoblasts to produce MMP-1 in high amounts and the stimulation of osteoblastic MMP-1 expression by myeloma cells was driven by p38. Additionally, the contact between myeloma cells and osteoblasts can significantly increase the ability of myeloma cells to invade and migrate. This indicates that the interaction between MMP-1 and p38/MAPK plays a crucial role in modulating the interplay between myeloma cells and BMSCs. The p38/MAPK pathway is a significant signaling cascade that participates in various cellular activities, such as growth, proliferation, differentiation, migration, and apoptosis (29, 30). In particular, the activation of the p38/MAPK pathway in myeloma cells increases their invasive and migratory abilities when they come into contact with BMSCs. In summary, the relationship between MMP-1 and p38/MAPK is critical for the investigation of the mechanisms of MM progression.

### 3.2 MMP-2 and MMP-9 in MM

MMP-2 and MMP-9 also referred to as gelatinase-A and gelatinase-B, are extensively expressed in almost all human tissues. Moreover, their functional architecture includes a fibronectin section within the catalytic domain (31). This extra fibronectin domain can be bound to and processed by denatured collagen or gelatine. These enzymes, according to research, not only degrade various ECM molecules, such as Collagen types I, IV, V, VII, X, IX, aggrecan, fibronectin, laminin, vitronectin, and elastin, but also break down non-ECM molecules like pro-TNF-α, pro-IL-β, pro-IL-8, TGF-β, and monocyte chemoattractant protein-3 (32). Gelatinases are involved in the onset and progression of a variety of human diseases, the most widely studied of these is diabetes mellitus (33, 34). In addition, gelatinase has been reported to be one of the key ECM-degrading enzymes involved in tumor invasion and metastasis (35). Based on this, we will conduct a more comprehensive analysis and discussion on the exact roles of MMP-2 and MMP-9 in multiple myeloma, as well as their potential clinical implications.

There have been numerous studies on the expression of MMP-2 and MMP-9 in MM patients during the past two decades. Vacca et al. (36) showed that active MM patients express significantly higher levels of MMP-2 mRNA and protein compared to inactive MM and MGUS patients by *in situ* hybridizations of BM PCs and gelatin-zymography, while MMP-9 expression was similar in all groups. Furthermore, immunoassays of plasma cell extracts showed that levels of angiogenic basic fibroblast growth factor (FGF) - 2 were significantly higher in active MM patients than in inactive MM patients and MGUS patients. These findings suggest that the angiogenic and invasive potential of MM is partially dependent on FGF-2 and MMP-2 production. In addition, Marquez-Curtis et al. (37) established Long-term Marrow Cultures (LTMCs) system from acute myelogenous leukemia (AML) patients and normal donors BM cells. The LTMCs system contains multiple BM cells, including bone marrow stromal cells (BMSCs) and progenitor cells, and can maintain the growth and differentiation of various BM cells *in vitro* for a long period. Supernatants from this system were collected for enzyme spectrum analysis, and analysis of normal LTMC culture results showed that proMMP-9 concentration gradually decreased with the increase of culture time, reaching the lowest level by the 4 ± 5th week, while proMMP-2 concentration gradually increased with the increase of culture time. However, compared with normal LTMCs, AML LTMCs displayed higher levels of proMMP-9. Zdzisińska et al. (27) co-cultured RPMI8226 cells with BMSCs obtained from MM patients and healthy controls, and found that MM patient-derived BMSCs produced significantly higher levels of MMP-2 compared to BMSCs from healthy controls, while RPMI8226 cells alone did not produce detectable levels of MMP-2. Van et al. (38) established an MM mouse model to mimic the growth and progression of human multiple myeloma by transplanting 5T33MM cells into mice, and their study found that 5T33MM cells secreted MMP-9 *in vivo*. Specifically, upon injection of 5T33MMvt cells into immature mice, MMP-9 secretion was upregulated in MM cells isolated from the BM (5T33MMvt-vv) during tumor development. However, when these cells were re-cultivated *in vitro*, MMP-9 production declined and was eventually eliminated, suggesting that the production of MMP-9 was controlled by the BM microenvironment. Notably, *in vitro*, interaction of 5T33MM cells with BM endothelial cells resulted in upregulation of MMP-9 in the 5T33MM cells. These studies have shown that although MMP-2 and MMP-9 share similar structures and general functions, their expression in MM is vastly different. In MM, MMP-2 is mainly produced by BMSCs, whereas MMP-9 is primarily produced by malignant PCs, and its production is regulated by the BM microenvironment (27, 38).

#### 3.2.1 MMP-2 and MMP-9 in myeloma bone disease

The mechanism of bone destruction induced by myeloma is that the increased activity of osteoclasts leads to excessive bone resorption, but there is no corresponding increase in osteogenesis, and thus bone formation is weakened (39). Gelatinases (MMP-2 and MMP-9) can combine with a variety of substrates such as collagens type I and IV, procollagen type II, etc (40), among them, collagen type I constitutes the bone collagen and not only provides a structural site for osteocalcin but also combines with non-collagenous proteins such as osteocalcin to form a network scaffold that provides essential conditions for bone mineralization (41), thus gelatinase is very significant for the progression of myeloma bone disease.

Sfiridaki et al. (42) confirmed that MMP-9 serum levels were considerably lower in MM patients compared to healthy controls, and according to the Durie Salmon stage, the average serum concentration of MMP-9 in the stage II patients was significantly higher than that in the stage I patients. Stage I MM patients are characterized by a hemoglobin level over 100g/L, normal serum calcium levels (≤3.0mmol/L or 12mg/dL), normal bone structure or solitary plasmacytoma on X-ray, low M protein levels (IgG<50g/L, IgA<30g/L, Bence Jones protein<4g/24h urine), and tumor cell count less than 0.6×10^12^/m^2^ body surface area. Stage II patients with the progressive disease have a tumor cell count of approximately 0.6 to 1.2 × 10^12^/m^2^ body surface area and develop osteolytic lesions (43). Moreover, Sfiridaki et al. (42) observed that plasma cell infiltration was positively correlated with bone resorption marker N-telopeptide (NTx) level and serum MMP-9. Notably, the average serum concentration of MMP-9 in stage I myeloma patients was lower than that in healthy controls, while it was higher in stage II patients compared to those in stage I. This difference may arise from the fact that MMP-9 in the serum of healthy individuals is primarily derived from circulating leukocytes. However, in stage I patients, the growth of normal blood cells is inhibited due to the proliferation of malignant plasma cells, leading to a decrease in MMP-9 levels. As the disease progresses, malignant plasma cells continue to proliferate and independently produce MMP-9. Consequently, stage II patients exhibit higher levels of MMP-9 than stage I patients.

#### 3.2.2 MMP-2 and MMP-9 in angiogenesis and metastasis

Due to the growth disorder of tumor cells and the tendency of distant invasion, the dynamic nature, and dysregulated growth of the tumor, the steady state of ECM is destroyed at the biochemical, biological, and structural levels (24). MMP is one of the major matrix-degrading enzymes, which can impact the overall integrity of the ECM by cleaving ECM and releasing cytokines, growth factors, and their receptors that bind to the cell surface (44, 45). MMP-2 and MMP-9 promote invasion by triggering the degradation of gelatin and type IV, V, XI, and XVI collagens which are vital for cellular invasion (25).

Angiogenesis is a crucial step in the development of cancer because tumors need a sufficient blood supply to meet the high demand for energy and nutrients for their multiplication (46). In the BM microenvironment of MM patients, microvessel density (MVD), endothelial activity, capillary permeability, as well as perfusion are enhanced, and the increase in MVD is triggered by oncogene-mediated expression and secretion of cytokines and proangiogenic growth factors (47). Studies on circulating PCs in MM have shown that higher BM-MVD correlates with the presence of circulating PCs but not with the rate of PC infiltration in the BM, implying that angiogenesis may accelerate PC proliferation and migration into the peripheral circulation (48).

Lamanuzzi et al. (49) found that the levels of BM and circulating thrombopoietin (TPO) in MM patients at different stages of progression were higher than those in MGUS/SMM patients, and both endothelial cells (MGECs) from MGUS patients and endothelial cells (MMECs) from MM patients expressed TPO receptors. Exposure to TPO *in vitro* increased the release of MMP-9 and MMP-2 in MGECs, as well as the release of MMP-2 in MMECs. Moreover, TPO was found to influence the balance between angiogenic and anti-angiogenic factors in the BM of MM and trigger angiogenesis. Additionally, several growth factors that influence angiogenesis, such as transforming growth factor- β (TGF-β) and vascular endothelial growth factor (VEGF) are exposed after the ECM is degraded by MMP-2 and MMP-9 (50). TGF-β, belonging to the transforming growth factor superfamily, plays different roles in a variety of biological processes and is considered a multifunctional cytokine (51). TGF-β forms a complex with latent-associated peptide (LAP), which renders TGF-β biologically inactive and in a latent state. The biological activity of TGF-β is only displayed when it is released from the complex. Therefore, this complex serves as a critical protective mechanism for cells, ensuring that TGF-β is only activated when needed (52). MMP-2 and MT1-MMP can activate TGF-β by releasing it from LAP (53, 54). Moreover, the TGF-β-LAP complex remains bound to the ECM by interacting with TGF-β binding protein (LTBP). MMP-9 can cleave soluble LTBP as well as the ECM-bound LTBP to activate TGF-β, but MMP-2 can only cleave soluble LTBP (55). MMP-2 and MMP-9 are elevated in angiogenic lesions because they can also activate VEGF, promoting vascular permeability and angiogenesis (56). Moreover, *in vitro* studies have shown that MMP-9 can increase the release of VEGF in normal pancreatic cells. Conversely, treatment with MMP inhibitors or knockdown of *MMP-9* in cells was found to reduce angiogenesis (56).

MMPs, on the other hand, may inhibit angiogenesis. MMP-2 and MMP-9, in particular, aid in the digestion of plasminogen and the release of angiostatin, which increases tumor cell apoptosis, and MMP-9 also breaks down collagen XVIII to produce endostatin, which inhibits angiogenesis (25).

### 3.3 MMP-7 in MM

In its physiological state, MMP-7 is mainly expressed in the human reproductive system, salivary glands, and prostate (57). Previous research has demonstrated that MMP-7 promotes *in vivo* osteolysis and is differently expressed in the tumor-bone microenvironment in breast and prostate cancer (58, 59). In MM, Barillé et al. (60) discovered that MMP-7 was able to stimulate BMSCs to produce MMP-2, which can degrade collagen I to promote bone absorption. Later, research by Thioloy et al. (58) demonstrated that MMP-7 produced from osteoclasts greatly aided tumor growth and tumor-induced osteolysis. All of their findings point to the possibility that blocking MMP activity might be a potential therapeutic approach for MM.

Nevertheless, another study soon after reached a completely different conclusion. In the study by Lwin et al. (61), a previously unknown role for MMP-7 in the pathogenesis of myeloma was discovered. The study found that MMP-7-deficient myeloma-bearing mice had a significantly higher tumor load and osteolytic bone disease compared to their wild-type counterparts, and clinical evidence supported the *in vivo* murine myeloma studies as well, which demonstrated a marked decrease in MMP-7 activity observed in MM patients. According to previous findings, MMP-7 is abundantly expressed by osteoclasts and able to cleave receptor activator for nuclear factor-κB (RANKL) expressed by osteoblasts can activate osteoclasts to a soluble active form (59). However, the data from Lwin et al.’s investigation indicate that there was no discernible change in RANKL levels between myeloma-bearing mice lacking MMP-7 and the wild-type control. Interestingly, they found an increase in myeloma cell viability by co-culturing with 2T3 pre-osteoblasts, whereas overexpression of MMP-7 in pre-osteoblasts suppressed this phenomenon (61). It follows that the promotion of osteolytic bone disease by loss of host-derived MMP-7 is not determined by a single mechanism.

The above study implies that rather than exerting a direct antitumor effect, MMP-7 achieves either a tumor-promoting or an inhibitory effect through a variety of complex mechanisms in the specific tumor microenvironment. Furthermore, interactions between MMP-7 and other signaling molecules or proteins might differ across studies, contributing to contrasting conclusions.

### 3.4 MMP-13 in MM

MMP-13 is predominantly expressed in the lung, skin, prostate, small intestine, breast, testis, and bladder in normal humans, but more importantly, it is widely expressed in the mesenchymal stromal cell (MSC) lineage, including chondrocytes, osteoblasts, and osteocytes; however, osteoclasts do not appear to express MMP-13 (57, 62). To date, MMP-13 participates in the progress of MM mainly through its proteolytic activity and catalytic activity (62, 63). Many of the important functional roles of MMP-13 have been directly connected to its capacity to breakdown interstitial collagen which is a critical structural component of all connective tissues, including bone, and other matrix-associated targets (64, 65). However, Fu et al. (63) demonstrate that MMP-13 expression is induced by IL-6-mediated interactions between MM and BMSC and acts as a potent osteoclastogenic factor that can encourage the production of multinucleated, bone-resorbing osteoclasts. Different from the previous mechanism, MMP-13 exerts its pro-osteoclastogenic effects without a requirement for proteolytic activity by acting as a secretagogue inducing DC-STAMP, which is an important fusogenic factor whose absence impairs osteoclasts formation and function (63). Myeloma-bone interaction is a vicious loop that enhances the bioavailability of cytokines and growth hormones, which promote tumor growth and increase therapeutic resistance. Surprisingly, soluble factor analysis from MMP-13-null mice revealed decreased bioavailability of various osteoclastogenic factors of MSCs including CXCL7 which is capable to promote the recruitment and formation of osteoclast precursors. Therefore, CXCL7 can be regarded as a new MMP-13 substrate and osteoclast production regulator. Moreover, this effect is determined by MMP-13 catalytic activity rather than proteolytic activity since the selective inhibitor of MMP-13 could obviously prolong overall survival in MM-bearing mice (62, 66).

### 3.5 MT-MMPs in MM

MT-MMPs are expressed almost in various tissues of the human urinary, reproductive, circulatory, and digestive systems in physiological status (57). MT-MMPs are a special subtype that can undergo pericellular proteolysis, which is considered an essential phase in the restructuring of paracancerous tissues (67). Among MT-MMPs, it has been reported that MT-MMP1 and MT-MMP2, also known as MMP14 and MMP-15, are mainly involved in MM progression (68–70). According to some research, MT1-MMP inhibits macrophage invasion by hydrolyzing ECM elements and cell surface chemicals and by triggering signal transduction pathways that affect motility and energy consumption (71). Several ECM proteins, including gelatin, fibronectin, laminin, and fibrillary collagens, are degraded by MT1-MMP and MT2-MMP, which can activate the pro-MMP2 on the cell surface as well (67, 72, 73).

MT1-MMP(MMP-14), the first membrane-type MMP discovered, was identified in 1994 as an activator of pro-MMP-2 (74). In MM, MMP-T1 has been confirmed to be expressed by malignant PCs and be conducive to CXCL12 promoting the invasion of myeloma cells via matrigel-reconstituted basal membranes and type I collagen gels (68). Several years later, Shimizu-Hirota et al. (71) identified in addition to acting as an ECM-degrading enzyme, MT-MMP1 also unexpectedly regulates inflammatory responses. They found that MT1-MMP^–/–^ macrophages produce excessive chemokine and cytokine responses to *in vitro* and *in vivo* immune stimuli. These researches prove that the role of MT-MMP1 in CXCL-12 promoting MM cells to invade the basement membrane may be related to macrophage immune response.

MT2-MMP (MMP-15), which shares 73.9% of its overall similarity with MT1-MMP, was initially identified from the human lung cDNA library (67, 75). It has been demonstrated that the human fibrosarcoma cell HT1080 expresses MT2-MMP. Moreover, it was shown that the migration and invasion of cancer cells were decreased following siRNA induction, which inhibited the endogenous expression of MT2-MMP (67). In comparison to B cell lines and other normal peripheral blood or BM-derived cells, the expression of MT2-MMP is markedly higher in MM cells, according to a recent study (70). When MT2-MMP was inhibited in U266 cells utilizing siRNA technology, it turned out to be the case that MT2-MMP siRNA considerably reduced its adhesion, invasion, migration, and proliferation capabilities (70). Taken together, the studies discussed above suggest that MT2-MMP may serve as a viable biomarker for both diagnosing and treating MM.

## 4 The role of TIMPs in MM

TIMPs have been found to regulate processes such as cell function, angiogenesis, apoptosis, cell differentiation, growth, and migration through both metalloproteinase-dependent and metalloproteinase-independent mechanisms (18).

### 4.1 TIMP-1 in MM

TIMP1 was first discovered and defined in 1972, which constitutively expresses in many mammalian tissues (16, 76). Nowadays TIMP1 has been found in a lot of tissues, including the heart, brain, colon, arteries, liver, kidneys, lungs, bladder, breasts, skin, lymph nodes, ovaries, uterus, prostate, and testes (57). Proinflammatory cytokines such as tumor necrosis factor-alpha (TNF-α) and interleukin-1beta(IL-1β) can increase the production of *TIMP1* mRNA (77). TIMP1 has a certain correlation with cell migration. For example, TIMP1 regulates the MMP-mediated degradation of vascular endothelial cadherin (VE-cadherin) at intercellular junctions and thereby inhibits microvascular endothelial cell migration (78). In addition, TIMP1 seemed to mediate bronchiole epithelial cell migration after injury by inhibiting the MMP7-dependent cleavage of syndecan-1, a heparan sulfate glycoprotein, whose shedding is associated with increased cell migration (79, 80). TIMP-1 inhibits many types of MMPs, including MMP-1, MMP-2, MMP-3, MMP-7, MMP-8, and MMP-9, it is worth mentioning that it exhibits a high binding affinity to both pro-MMP-9 and active MMP-9 and has been seen as the most effective inhibitor of MMP-7 in four TIMP families (57, 81). Of note, significantly elevated TIMPI levels were consistently found in both mice and patients with MM bone disease, while MMP-7 levels decreased (61). In addition, it has been observed that human MSCs are capable of secreting TIMP-1, which aids in reducing the levels of MMP-9 produced by MM cells and consequently prevents their migration (82). Furthermore, interactions between TIMP-1 and the hemopexin domain of pro-MMP-9 lead to the formation of a compound that hinders MMP-9 activation, which in turn inhibits the production of MMP-9 (83). These phenomena also indicate that TIMP1 has the potential to be a novel therapeutic target for MM and osteolytic bone disease.

### 4.2 TIMP-2 in MM

The cloning and sequencing of TIMP-2 were initially reported in 1990, wherein the cDNA library of A2058 human melanoma cells was utilized for this purpose (84). More importantly, in 1991, it was demonstrated that TIMP-2 functions as a potent inhibitor of collagenase activity by forming a stable complex with a 1:1 molar ratio between TIMP-2 and the enzyme (85). The lymph nodes, brain, heart, arteries, colon, kidneys, liver, breasts, ovaries, prostate, and testes are among the tissues that express TIMP-2 (57). Previous studies (57, 86) have shown that TIMP-2 also regulates MT1-MMP, MMP-2, and MMP-9 activity. Furthermore, a recent study has confirmed a correlation between lower expression of TIMP-2 and/or higher expression of MMP-9 in colon cancer tissues and poorer overall survival. Additionally, both *in vitro* and *in vivo* experiments have shown that TIMP-2 efficiently prevents the invasion and migration of HCT 116 cells through the regulation of MMP-9 (87). In non-small cell lung cancer(NSCLC), TIMP-2, as a protective factor, is up-regulated and related to the prognosis (88). A recent study has confirmed that miR-483-5p exerts a tumorigenic effect in MM by targeting TIMP-2, leading to its attenuation in MM cells, which in turn promotes cell proliferation and inhibits apoptosis, thereby facilitating MM progression (89).

### 4.3 TIMP-3 in MM

TIMP-3 was cloned in chickens and humans in 1992 and 1994, respectively (90–92). TIMP-3 expression has been detected in various tissues, including the brain, heart, colon, kidneys, lungs, liver, breasts, ovaries, prostate, and testes (57). Mechanistically, TIMP-3 has been shown to function as a potent activator of apoptotic pathways by inhibiting the release of death receptors like Fas from the cell surface, which is mediated by its N-terminal domain, thereby promoting the cleavage and activation of downstream apoptotic signaling molecules (93). Given that TIMP-3 is not secreted like the other three TIMP families but rather exists in the matrix, it is not unexpected that TIMP3 can affect ECM-cell signaling mediated focal adhesion kinase (FAK) and fibronectin (FN) (94). TIMP-3 is associated with a variety of cancers, including kidney cancer and brain cancer, and the methylation-related silencing of the TIMP-3 gene indicates its inhibitory effect on cancer progression (95). Besides that, its N-terminal domain can interact with the active sites of MMP-2 and MMP-9 to exert its MMP inhibitory effect (57). It is well known that Interleukin-6 (IL-6) is the main growth factor of human myeloma cells, which acts through its receptor IL-6R. *In vivo*, IL-6R is expressed on the surface of myeloma cells and released into the serum to become a soluble form (sIL-6R). Clinical research has suggested that the level of serum IL-6R correlates with the poor prognosis of patients. Importantly, TIMP-3 and BB-94, a metalloproteinase inhibitor based on hydroxamic acid, can prevent the release of sIL-6R. In other words, TIMP-3 mediated inhibition of sIL-6R release may be one of the methods for treatment in MM (96).

### 4.4 TIMP-4 in MM

TIMP-4 was cloned from a human heart cDNA library by using the expressed sequence tag sequencing approach in 1996 (97). It has been discovered in several organs and tissues, including the brain, heart, kidneys, breasts, uterus, pancreas, colon, ovaries, testes, prostate, and adipose tissue (57). TIMP-4 is the main MMP inhibitor in human platelets, and it affects platelet recruitment and aggregation (98). In addition, TIMP-4 has also been known to regulate the activity of MMP-2 by acting as the negative regulator of MT1-MMP (99). As mentioned above, MT1-MMP and MMP-2 are closely related to MM and osteolytic osteopathy.

## 5 Inhibitors of MMP

Due to its important role in the progression of various diseases, including multiple myeloma, MMP has become a crucial target for disease diagnosis and treatment. Over the years, numerous naturally derived or artificially designed MMP inhibitors have been gradually developed.

In terms of material design, MMP inhibitors have undergone three generations of development, from the first generation of hydroxamate-based peptidomimetics, to the second generation of non-hydroxamate compounds, and finally to the third generation of nanomaterials (100). In recent years, there is increasing evidence that nanomaterials may play a role in regulating MMP activity. However, most research has only focused on changes in MMP activity induced by nanomaterials, while the understanding of the underlying mechanisms remains limited. Based on current studies, the main reasons for changes in MMP activity are gene mutations, protein expression alterations, and direct activity inhibition (100). Hashimoto et al. (101) found that Gold Nanoparticles (AuNPs) can inhibit the activity of MMPs, and this inhibitory effect depends on the size and surface charge of the AuNPs. Smaller AuNPs are able to effectively inhibit MMP-2 and -9. AuNPs with negative charges chelate the active site Zn^2+^ of MMPs, thereby inhibiting MMP-2 and -9. However, the regulatory effects of nanomaterials may vary in different cell lines. Due to the tunability of nanoparticle properties, fine-tuning the characteristics of enables them to safely and effectively enter biological systems, providing an opportunity for further optimization of their biological functions. In terms of drug screening and design, with the development of computer simulation-assisted design and complex biochemical techniques such as antibody and protein engineering, the design of MMP inhibitors has gradually shifted from broad-spectrum inhibitory activity to targeting specific MMP inhibitory activity. Although some early synthesized drugs showed significant effects *in vitro*, their oral bioavailability was poor, and they were accompanied by many side effects. Some later synthesized selective inhibitors, although performed well in preclinical stages, showed poor efficacy in clinical trials, which may be related to specific tumor microenvironments (100).

## 6 Conclusion

MM remains one of the most lethal cancers despite recent advancements in treatment. Patients with MM also experience systemic skeletal lesions that significantly diminish their quality of life. MMPs and TIMPs have been implicated in MM metastasis, angiogenesis, and extensive bone destruction, but the exact molecular mechanisms remain unknown. Although tissue inhibitors of metalloproteinases (TIMPs) have great theoretical potential as a novel class of metalloproteinase inhibitors and can be developed into new drugs, in reality, they have not received sufficient attention and application due to the possibility that TIMPs may inhibit the activity of matrix metalloproteinases (MMPs) while also carrying risks of directly inhibiting or indirectly promoting the activity of MMPs. Additionally, the complex tumor microenvironment regulation by numerous enzymes and cytokines, along with the diverse functions of various MMPs and TIMPs in different tumor types, present a major challenge in developing specific MMP inhibitors. Therefore, in order to better understand the specific roles of MMPs and TIMPs in MM and to improve their therapeutic efficacy, further basic research is needed. Furthermore, investigating their mechanisms of action can provide more evidence for their potential as biomarkers and predictors of disease progression.

## Author contributions

All five authors Y-YL, L-YZ, Y-HX, DL and JZ jointly wrote, reviewed, and edited the article. All authors contributed to the article and approved the submitted version.
