# Supplementary material for: Matrix metalloproteinases and tissue inhibitors in multiple myeloma: promote or inhibit?
Source: Front Oncol. 2023 Sep 26;13:1127407. doi: 10.3389/fonc.2023.1127407 (PMC10562598; doi:10.3389/fonc.2023.1127407)
Supplement: Supplementary file 1 [file DataSheet_1.docx]

Supplementary Material

Matrix metalloproteinases and tissue inhibitors in multiple myeloma: promote or inhibit?

Li Yan-Ying, Zhang Liu-Yun, Xiang Yun-Hui, Dan Li^*^，Zhang Juan*

*** Correspondence:** Zhang Juan: zhangjuan82@126.com

# Supplementary Figures and Tables

## Supplementary Figures


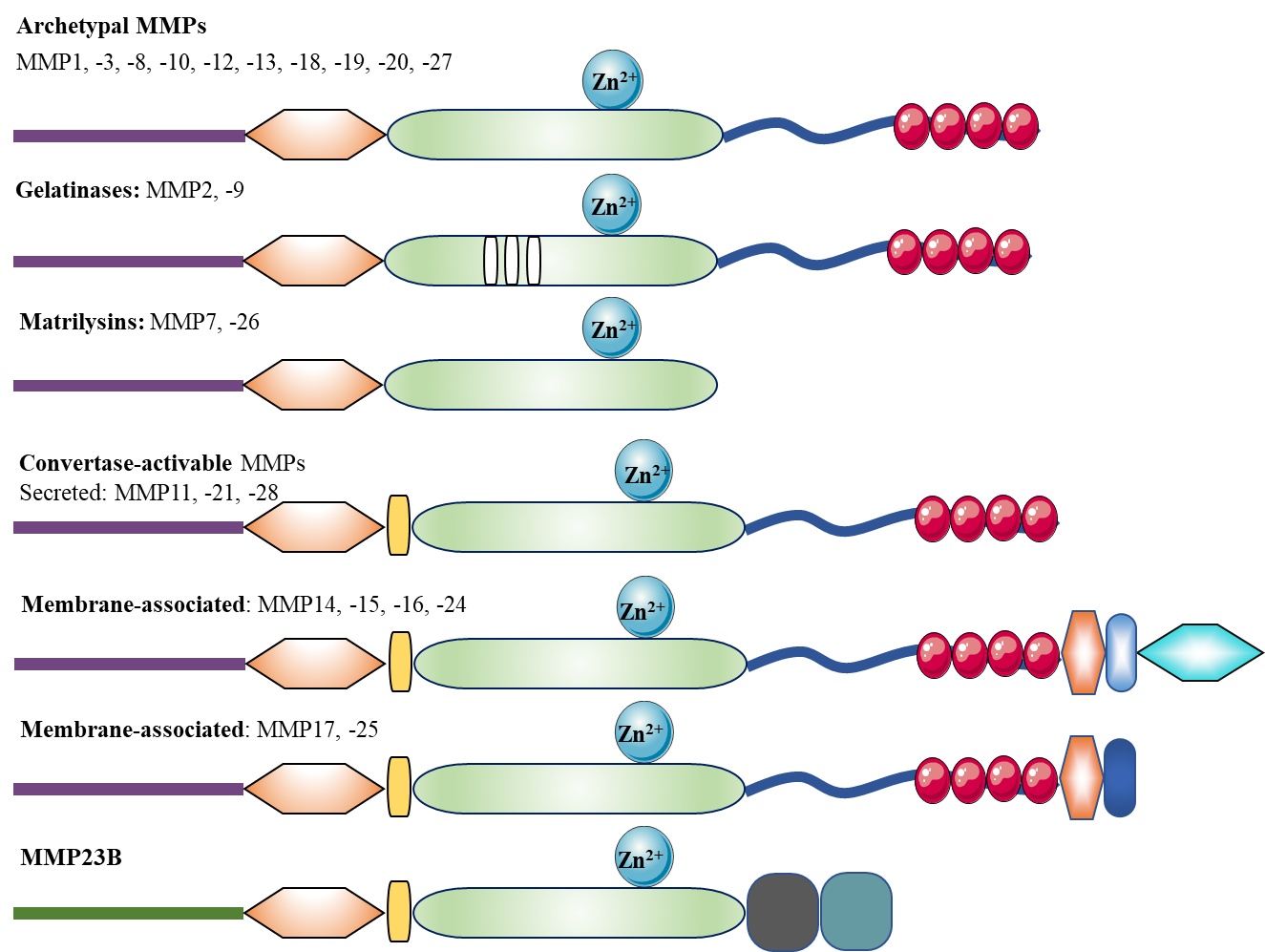


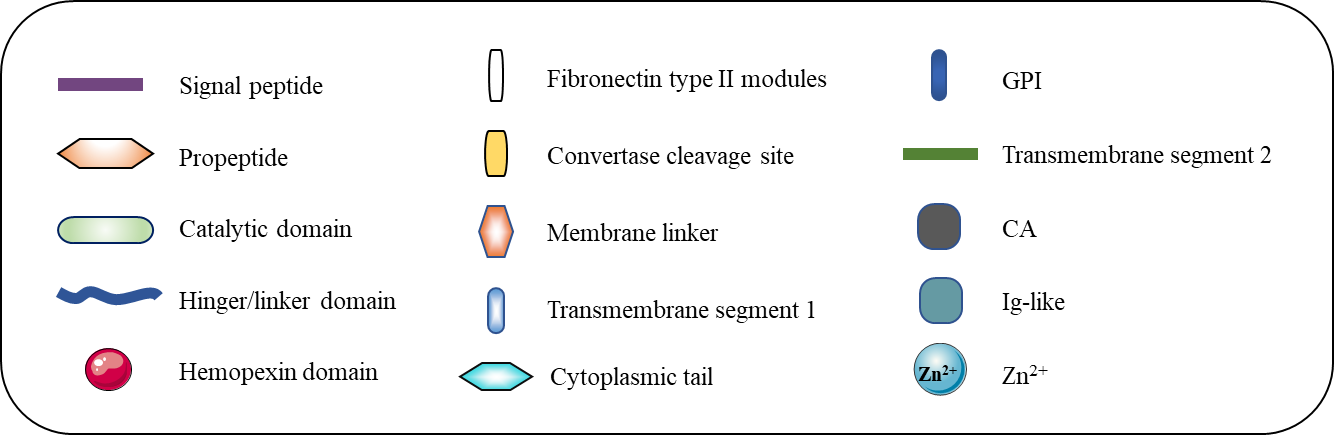


FIGURE 1

Schematic diagram of different domains of 23 human MMPs. The archetypal MMPs contain a signal peptide, a propeptide, a catalytic domain that binds a Zn2+ residue, and a hemopexin C-terminal domain. Compared to archetypal MMPs, gelatinases contain a fibronectin type-II modules domain, matrilysins lack the hemopexin C-terminal domain, and convertase-activable MMPs contain a convertase cleavage site. In addition to invertase cleavage sites, a membrane linker is also contained by membrane-type MMPs, which are divided into two categories, one contains a transmembrane segment 1 and a cytoplasmic tail, and another contains GPI. MMP-23B has no linker domain but contains a cysteine array (CA) and an immunoglobin-like (Ig-like) domain.


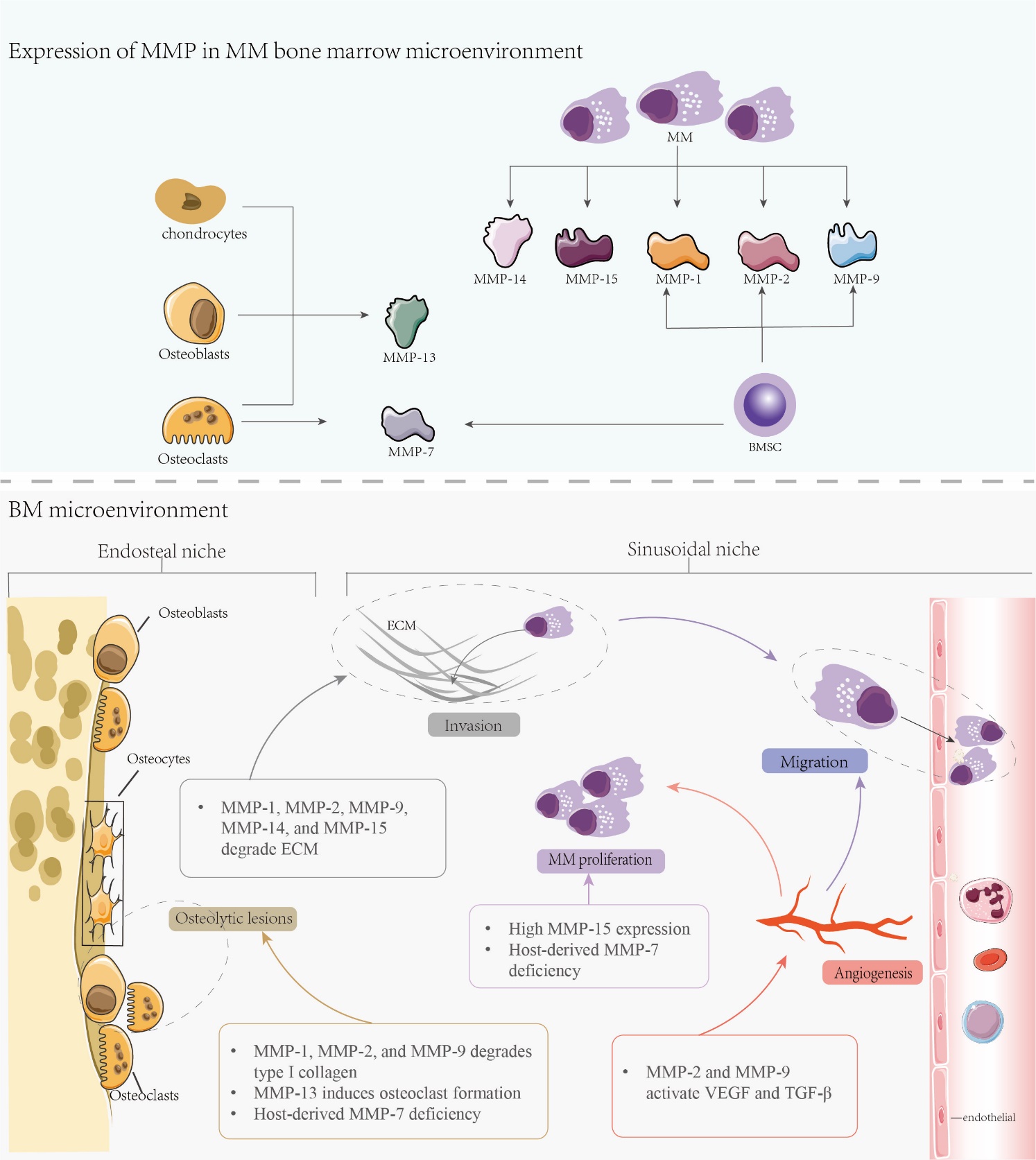


FIGURE 2

The expression of MMP in the MM bone marrow microenvironment and the role of MMPs in MM initiation and progression. MMP-1 can be produced by BMSCs and MM cells and degrade type I collagen and induce bone resorption. MMP-2 and MMP-9, which are produced by BMSCs and MM cells, can degrade ECM and activate TGF-β and are involved in tumor invasion, metastasis, and angiogenesis. Both BMSCs and MM cells express MMP-7, which serves two functions: first, it encourages BMSCs to produce MMP-2, and second, it appears to suppress the proliferation of MM cells. MMP-13, expressed by chondrocytes, osteoblasts, and osteocytes, can break down the ECM involved in tumoral invasion. Both MMP-14 and MMP-15 are expressed by malignant plasma cells and favor the invasion of MM cells; the former induces the penetration of myeloma cells across the ECM via CXCL-12 and is related to the macrophage immune response.
